# Energy dependence of the response of X-ray multimeter for radiation qualities in mammography

**DOI:** 10.1038/s41598-025-93485-5

**Published:** 2025-03-21

**Authors:** Elisabeth Salomon, Peter Homolka, Istvan Csete, Paula Toroi

**Affiliations:** 1 Center for Medical Physics and Biomedical Engineering, Medical University of Vienna, 1090 Vienna, Austria; 2 Section of Dosimetry and Medical Radiation Physics, International Atomic Energy Agency, 1220 Vienna, Austria; 3 Radiation and Nuclear Safety Authority, STUK, 01370 Vantaa, Finland

## Abstract

Ensuring comprehensive quality control of breast imaging systems involving ionizing radiation like mammography and tomosynthesis is crucial for high diagnostic confidence and maintaining an acceptable patient dose. This requires accurate dosimetric measurements, including air kerma, half-value layer (HVL), and tube voltage as key quantities. Ionization chambers or semiconductor-based X-ray multimeters (XMMs) are used to measure these parameters, with XMMs also displaying tube voltage in one exposure in addition to numerous other parameters. To correct for the influence of slight changes in the X-ray spectra on the response of XMMs, dedicated algorithms are implemented in the XMMs’ software. They often require manual selection of anode/filter combinations prior the measurements. However, to ensure comparability, consistency, and traceability, measurement equipment must be calibrated for each specific measurement quantity. National dosimetry laboratories may have limited options for calibration, and errors can occur if the wrong combination is selected in the XMM software. This study investigates the hypothesis that the selection of the anode/filter combination in XMM software influences the readings. The primary objective is to evaluate the impact of different anode/filter combinations selected in the software on the measurement of air kerma, half-value layer (HVL), and tube voltage. Additionally, the study assesses the feasibility of performing quality assurance for XMMs using a limited range of anode/filter combinations. The readings of eight commercially available XMMs for air kerma rate, HVL and tube voltage were compared with the reference values realized in the IAEA secondary standards dosimetry laboratory for five anode/filter combinations and tube voltages ranging from 25 to 35 kV. The deviation of XMMs readings with different selections of anode/filter combinations in the software was studied. The maximum deviation when anode/filter combination selected in the XMM software matched the anode/filter combination of the X-ray beam was 19% for air kerma, 9% for tube voltage and 10% for HVL. When the selected anode/filter combination set differed from the one used, maximum deviation increased up to 31% for air kerma, 44% for tube voltage and 45% for HVL. Appropriate selection of the anode/filter combination in the XMM software is crucial for obtaining reliable measurement results. Interpolation of calibration coefficients between different radiation qualities and selections is not recommended.

## Introduction

Ensuring comprehensive quality control of X-ray systems used for imaging procedures involving ionizing radiation is essential for achieving high diagnostic confidence while maintaining an acceptable dose to the patient. Patient-specific optimization of radiation quality, defining the spectra and the evaluation of individual risk for radiation-induced cancer are now standard practices in personalized medicine. These practices are predicated on accurate dosimetric measurements, with key parameters including air kerma and radiation quality specifiers as half-value layer (HVL) and tube voltage. The advent of digital imaging detectors and new imaging techniques, such as volumetric imaging, has expanded the variety of available radiation qualities. Especially in breast imaging modalities like mammography, tomosynthesis or contrast enhanced mammography, there is a wide range of anode-filter combinations at various tube voltage levels used in clinical practice.

The diversity of available radiation qualities presents a challenge for dosimetry equipment, that must not compromise the accuracy of dosimetric measurements including regular quality control. In patient-related dosimetry for mammography, the primary quantity of interest is the mean glandular dose (MGD, also termed average glandular dose, AGD). Direct measurement of MGD is not feasible; thus, tabulated factors are employed to convert incident air kerma to MGD^1–6^. These conversion factors are derived using Monte Carlo simulations and are tabulated based on the anode-filter combination, half-value layer (HVL) and compressed breast thickness. Consequently, precise measurements of air kerma and HVL are essential.

The European and IAEA quality assurance guidelines^7,8^ prescribe tube voltage measurements for the acceptance test. In addition, the European guidelines^7^ recommend that a tube voltage measurement be carried out every six months. On the other hand, the Mammo Protocol released by the European Federation of Organisations for Medical Physics (EFOMP)^9^ discarded the test of tube voltage measurement from the quality control protocol because of the modest effect on the final processed image. However, it should be kept in mind, that unintended change of tube voltage is still a serious sign of malfunction, and direct tube voltage measurement would provide a more direct indication of this incident than an HVL measurement. In the EFOMP Protocol for quality control in digital breast tomosynthesis^10^ check of accuracy of tube voltage is still recommended using a calibrated XMM for acceptance and routine testing of the system.

These quality control measurements are carried out either with an ionization chamber to determine air kerma and HVL or with a semiconductor-based X-ray multimeter (XMM), which offers the advantage of also displaying the tube voltage with just one exposure in addition to the aforementioned quantities. In contrast to ionization chambers, whose response is robust to slight changes in the X-ray spectra, the response of semiconductor-based detectors shows a pronounced energy dependence. To correct for this, dedicated algorithms are implemented in the XMMs’ software and the anode/filter/tube voltage range the XMMs is suitable for, is specified and must be adhered to. Most of the XMMs commercially available require a selection of the anode/filter combination prior to the measurements. This fact indicates that the selection of the anode/filter combination influences the displayed values. However, to ensure comparability, consistency, and traceability, measurement equipment must be calibrated for each specific measurement quantity corresponding to the relevant radiation quality^11^, but often the national dosimetry laboratories have only a limited set of radiation qualities for calibration and cannot cover all clinically relevant anode/filter combinations^12^. Most manufacturers of XMMs operate certified calibration laboratories, enabling users to send their devices for calibration and, if necessary, adjustment. However, logistic and organizational constraints often prevent healthcare providers from sending their equipment to the manufacturer located in another country. To maintain independence from the manufacturers of XMMs, it is essential for national calibration laboratories to be available for these calibrations. As a minimum, they should be able to perform constancy tests to verify the continued proper functioning of the device.

Further, the personnel responsible for carrying out quality assurance can face the situation that the anode/filter combination of the X-ray breast imaging system is not listed in the software of the XMM. In addition, a mistake can happen, and the user may select the wrong combination in the XMMs software for the measurement. In clinical practice, the calibration factor of XMMs is typically not applied directly during routine measurements. Instead, calibration is performed as part of quality control procedures, which are subject to predefined accuracy limits. The uncertainty introduced by the calibration process contributes to the overall uncertainty of these quality control measurements since it is the starting point for the uncertainty budget of the clinical measurements.

An extensive study of calibration coefficients was performed earlier using the correct selection of anode/filter combination^13^. However, this study is now extended to examine the impact of the anode/filter selection. By determining the calibration coefficient for different anode/filter combinations of the X-ray beam while keeping the anode/filter selection in the XMM software constant, we aim to gain a deeper understanding of how XMM algorithms respond to varying beam conditions. Determining the calibration coefficient for various anode/filter combinations selected in the XMM software, while maintaining a constant anode/filter combination in the X-ray beam, simulates a typical scenario where the radiation qualities available for calibration do not cover the possible selections within the XMM’s software. The current IEC 61267 standard only contains Mo/Mo as anode/filter combinations for determining the characteristic curve. This does not correspond to the broad spectrum of anode/filter combinations actually used in mammography. In order to cover a wider range of calibration conditions than provided in this standard, additional filters and a second anode material were included in this study.

The calibration of XMMs poses several challenges compared to the calibration of ionization chambers. For instance, each XMM requires the installation of specific software, with the preferred practice being the use of the same software version as employed by the end-user during measurements. Additionally, XMMs of later generations often incorporate software options for selecting not only various anode/filter combinations but also specific manufacturers or models of mammography systems. Furthermore, the X-ray system in the calibration facility may differ from the clinical system in parameters such as anode angle or dose rate. While previous studies demonstrated that such differences have a negligible impact on the calibration of ionization chambers, their influence on XMM calibration has yet to be fully established^14^. Addressing this issue falls outside the scope of the present work. Moreover, discrepancies may arise if the anode/filter combination selected in the XMM’s software is not available in the calibration laboratory. This study provides insights into whether limited calibration conditions can be effectively utilized for XMM measurements. The data aims to determine the feasibility of interpolating calibration coefficients between different radiation qualities based on specific radiation quality specifiers, i.e. whether it is possible to interpolate the calibration coefficients between radiation qualities with similar HVL but different spectra, such as W/Al and Mo/Mo or Mo/Rh. This data can be utilized to estimate errors and uncertainties in scenarios where the radiation quality selected differs from the actual beam. This helps us to understand the possibilities and constraints of using limited calibration data for a large range of clinical conditions.

## Materials and methods

### Measurement facility and reference standard

The XMMs included in this study where calibrated in accordance with IAEA TRS-457^11^ and as described in appendix to the calibration certificate^15^ at the dosimetry laboratory (DOL) of the IAEA. In accordance with the Minimum Reporting Standards^16^ the irradiation conditions are summarized as: All X-ray beam qualities were generated with a RTW MCD 100 H-5Mo (Mo-anode, RTW, Röntgen-Technik Dr. Warrikhoff GmbH, Neuenhagen bei Berlin, Germany) or an Isovolt MXR 160/0.4-3.0 (W-anode, Seifert Isovolt, Waygate Technologies, Hürth, Germany) X-ray tube, respectively. As high-voltage generator an ISOVOLT160 Titan E supplying a constant potential was applied, thus the resulting tube voltage (kV) corresponds to the tube peak voltage (kVp) and the practical peak voltage (PPV)^17^. High voltage output was monitored by a high-voltage divider, type FUG HVT 160000 (HV Technologies, Manassas, VA, USA), calibrated at PTB. The tube current (mA) was adjusted to obtain an air kerma rate of 50 mGy/min at a distance of 1 m from the focal spot. The field at the reference distance was circular with a diameter of 10 cm. The characteristics of the resulting X-ray beam qualities specified as filtration and HVL are summarized in Table 1. Both, the reference standard and the XMMs, were positioned free in the air, with a distance of the reference point set to 1 m from the focal spot. The reference conditions refer to a temperature of 20 °C, an air pressure of 101.325 kPa and a relative humidity of 50%. Deviations of the ambient conditions controlled by the air conditioning system from the reference temperature and air pressure have been taken into account by applying the correction factor for the reference standard^18^. XMMs specify a range of ambient conditions in their manual, and do not require manual correction.

**Table 1:** Characteristics of the X-ray beam radiation qualities used for calibration.

Anode/filter	Filter thickness in µm	HVL in mm Al			
		25 kV	28 kV	30 kV	35 kV
Mo/Mo	33	0.28	0.32	0.34	0.37
Mo/Rh	28.5	–	0.39	0.41	0.44
W/Al	513	0.32	0.36	0.39	0.44
W/Rh	47.7	0.46	0.49	0.50	0.54
W/Ag	49.2	0.47	0.52	0.54	0.59

The reference standard for the X-ray beam qualities used was a Radcal 10X5-6M ionization chamber (Radcal Cooperation, Monrovia, US) connected to a Keithley 6517A electrometer (Keithley Instruments, Solon, USA) traceable to the primary laboratory Bureau International des Poids et Mesures (BIPM, France) for Mo/Mo and Physikalisch-Technische Bundesanstalt (PTB, Germany) for all other radiation qualities. The electrometer was calibrated separately from the ionization chamber and the traceable calibration coefficient was applied to the reading. Over the range of the used radiation qualities the variation of the response of the ionization chamber is not more than ± 0.3%. For the measurements presented in this study an ionization chamber of the same type as the reference standard was used as a working standard. It has been calibrated against the reference standard prior to the measurements. The HVLs of the radiation qualities have been determined in a narrow beam, low scatter geometry as described in IAEA TRS-457^11^ using Al sheets with 99.99% purity in a diaphragm system positioned in half distance between the ionization chamber and the focal spot.

### X-ray multimeters

The X-ray Multimeters included in this study are described below. The focus of this work was to test a representative selection of XMMs from the most widely used manufacturers. Four of the selected XMMs represent the latest generation of XMMs from the respective manufacturer at the time of the measurements (Black Piranha, X2, AGMS-DM+, Nomex). The other four are XMMs of earlier generations that are still used in clinical practice. The selection was limited by the accessibility of the devices to the authors. The software used is part of the XMM and is already installed on the XMM for direct reading such as the Mult-O-Meter, Xi, X2 and Nomex. For meters without their own display unit, such as the R100B, Red Piranha, Black Piranha and AGMS-DM+, the corresponding software is supplied when the meter is purchased. Table 2 summarizes the software versions used as well as the maximum deviation stated by the manufacturer. Tables 3 and 4 show the measurement capabilities for air kerma, HVL and tube voltage and the possible selections of the radiation qualities in the software for the selected anode/filter combinations. The grayed out and crossed out entries in Table 3 indicate the XMMs that do not measure the corresponding quantity. In Table 4 the grayed out and crossed out entries represent the available selections in the software which have not been used for this study.

**Table 2:** Specifications of the XMMs.

Name	Measuring assembly	Manufacturer	Software	Max deviation		
				Dose/dose rate	HVL	Tube voltage
R100B	Barracuda	RTI, Möndal, Sweden	Ocean, Version: 2010.12.15.38	± 5%	–	–
Mult-O-Meter	Internal detector	Unfors, Billdal, Sweden	Display read directly	± 5%	–	–
Red Piranha	Internal detector	RTI	Ocean, Version: 2010.12.15.38	± 5%	–	± 2% or ± 1 kV
Black Piranha	Internal detector	RTI	Ocean, Version: 2016.06.14.214	± 5%	± 10%	± 2% or ± 1 kV
Xi	MAM	Unfors	Xi View, Version:3.0 (built47)	5%	± 5%	± 2% or ± 0.5 kV
X2	MAM	Unfors	X2 View, Version:1.7.8.0	5%	± 5%	± 2% or ± 0.5 kV
AGMS-DM+	Accu Gold	Radcal, Monrovia, CA, US	Accu-Gold by Radcal, Version 1.5.3.0 Driver 02.10.00	± 5%	± 10% (0.05 mmAl)	± 2% or ± 0.7 kV
Nomex	Nomex Multimeter	PTW, Freiburg, Germany	Nomex Software S03008,3.0	± 1%	± 0.01 mmAl	± 0.5 kV

**Table 3:** Measurement capabilities of the XMMs.

Anode/filter	Dose/dose rate		HVL		Tube voltage	
Mo/Mo	R100B	Xi		Xi		Xi^1^
	Mult-O-Meter	X2		X2		X2^2^
	Red Piranha	AGMS-DM+		AGMS-DM+	Red Piranha^1^	AGMS-DM+^2^
	Black Piranha	Nomex	Black Piranha	Nomex	Black Piranha^1^	Nomex^3^
Mo/Rh		Xi		Xi		Xi^1^
		X2		X2		X2^2,4^
	Red Piranha	AGMS-DM+		AGMS-DM+	Red Piranha^1^	AGMS-DM+^2^
	Black Piranha	Nomex	Black Piranha	Nomex	Black Piranha^1^	Nomex^3^
W/Al		Xi		Xi		Xi^1^
		X2		X2		X2^2^
	Red Piranha				Red Piranha^1^	
	Black Piranha	Nomex	Black Piranha	Nomex	Black Piranha^1^	Nomex^3^
W/Rh		Xi		Xi		Xi^1^
		X2		X2		X2^2^
	Red Piranha	AGMS-DM+		AGMS-DM+	Red Piranha^1^	AGMS-DM+^2^
	Black Piranha	Nomex	Black Piranha	Nomex	Black Piranha^1^	Nomex^3^
W/Ag		Xi		Xi		Xi^1^
		X2		X2		X2^2^
	Red Piranha	AGMS-DM+		AGMS-DM+	Red Piranha^1^	AGMS-DM+^2^
	Black Piranha	Nomex	Black Piranha	Nomex	Black Piranha^1^	Nomex^3^

^1^Tube voltage (kV), ^2^peak tube voltage (kVp), ^3^practical peak voltage (PPV), ^4^requires 2 mm additional Al filtration for tube voltage measurements.

**Table 4:** Available selections for the anode/filter combinations included in this study.

R100b	Mult-O-Meter	Red Piranha	Black Piranha	Xi	X2	AGMS-DM+^1^	Nomex
Mo/30 µm Mo	Mo/30 µm Mo	Mo/30 µm Mo	Mo/30 µm Mo	Mo/Mo	Mo/Mo	Mo/Mo	Mo/30 µm Mo
–	–	Mo/25 µm Rh	Mo/25 µm Rh	Mo/Rh	Mo/Rh	Mo/Rh	Mo/25 µm Rh
–	–	W/0.5 mm Al	W/0.5 mm Al	W/Al	W/Al	–	W/0.7 mm Al
–	–	W/50 µm Rh	W/50 µm Rh	W/Rh	W/Rh	W/Rh	W/50 µm Rh
–	–	W/55 µm Ag	W/50 µm Ag	W/Ag	W/Ag	W/Ag	W/50 µm Ag

^1^Reference Conditions with additional 2.2 mm polycarbonate.

### Calculation of the calibration coefficients

All calibrations have been performed using the substitution method as described in^11^. Measurements are first performed with the reference ionization chamber in the radiation field and after with the XMM to be calibrated. The calibration coefficient $${N}_{{X/X}_{b}{,Y/Y}_{s}}^{{\dot{K}}_{air},HVL,kV}$$ was calculated according to (1) were $$R$$ is the reference value for corresponding quantity air kerma rate $${\dot{K}}_{air}$$, HVL or tube voltage ($$kV)$$ and $${r}_{{X/X}_{b}{,Y/Y}_{s}}$$ the reading of the XMM. The indices $${X/X}_{b}$$ represent the anode/filter combination of the X-ray beam and $${Y/Y}_{s}$$ the anode/filter combination selected in the XMMs software. The reading of the XMM was determined as the average of three consecutive exposures of ten seconds each.

1 $${N}_{{X/X}_{b}{,Y/Y}_{s}}^{{\dot{K}}_{air},HVL,kV}=\frac{R}{{r}_{{X/X}_{b}{,Y/Y}_{s}}}$$

### Uncertainties

Measurement uncertainties were classified as type A (evaluated by statistical analysis) and type B (determined by other means) and assessed according to^19,20^. The expanded uncertainty (k = 2) for the reference value $R$ for air kerma rate determined by the working standard is 1.27%. The detailed uncertainty budget for this setup was already evaluated in our previous study and is provided in^13^. The major contributions to the uncertainty of the reference HVL are the thickness of the Al-sheets used, change in the spectra and interpolation for tube voltage it is mainly the calibration of the high voltage divider. For both quantities the resulting expanded uncertainty (k = 2) for reference value is not more than 1%^13^. The XMM’s contribution to the expanded uncertainty of the resulting calibration coefficient encompasses the uncertainties arising from positioning, as well as from reading. For positioning 0.03% (Type B) was derived^13^, the contribution of the XMM reading is estimated by the coefficient of variation in percent of three consecutive measurements individually for every XMM for ${X/X}_{b}=Y/{Y}_{s}$ and ${X/X}_{b}\ne Y/{Y}_{s}$ and summarized with the resulting expanded uncertainty in Table 5.

**Table 5:** Coefficient of variation (CV) in % for air kerma rate, HVL and tube voltage measurement and the corresponding expanded uncertainty U in % for k = 2 (in brackets) for the calibration coefficient of the X-ray multimeter.

	R100b	Mult-O-Meter	Red Piranha	Black Piranha	Xi	X2	AGMS-DM+	Nomex
${CV}_{{\dot{K}}_{air}}$%								
$\frac{X}{X}_{b}=\frac{Y}{{Y}_{s}}$	0.03 (1.27)	0.05 (1.28)	0.06 (1.28	0.09 (1.29)	0.10 (1.29)	0.11 (1.30)	0.05 (1.28)	0.00 (1.27)
$\frac{X}{X}_{b}\ne \frac{Y}{{Y}_{s}}$	0.02 (1.27)	0.07 (1.28)	0.47 (1.58)	0.12 (1.29)	0.13 (1.30)	0.10 (1.29)	0.06 (1.28)	0.00 (1.27)
${CV}_{kV}$ %								
$\frac{X}{X}_{b}=\frac{Y}{{Y}_{s}}$	–	–	0.13 (2.02)	0.10 (2.01)	0.06 (2.00)	0.19 (2.03)	0.18 (2.03)	0.16 (2.03)
$\frac{X}{X}_{b}\ne \frac{Y}{{Y}_{s}}$	–	–	0.13 (2.02)	0.09 (2.01)	0.33 (2.11)	0.26 (2.07)	0.22 (2.05)	0.14 (2.02)
${CV}_{HVL}$ %								
$\frac{X}{X}_{b}=\frac{Y}{{Y}_{s}}$	–	–	–	0.02 (2.00)	0.14 (2.02)	0.38 (2.14)	0.06 (2.00)	0.10 (2.02)
$\frac{X}{X}_{b}\ne \frac{Y}{{Y}_{s}}$	–	–	–	0.02 (2.00)	0.29 (2.08)	0.38 (2.14)	0.07 (2.00)	0.13 (2.02)

## Results

**Fig. 1:**
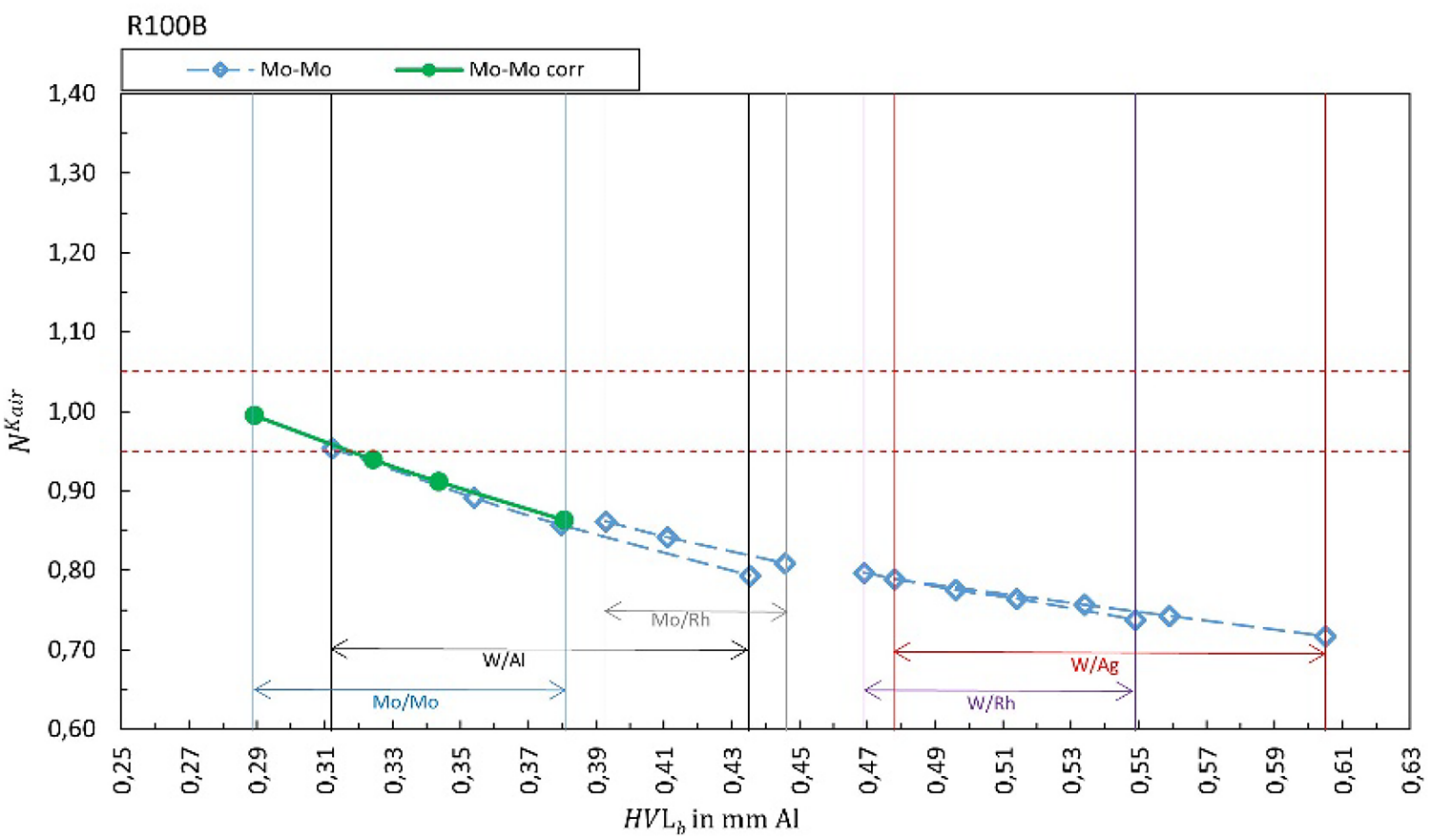
Calibration coefficient for air kerma rate for R100B. The solid green markers represent ${N}_{{X/X}_{b}{=Y/Y}_{s}}^{{\dot{K}}_{air}}$. Open markers represent the calibration coefficients ${N}_{{X/X}_{b}{\ne Y/Y}_{s}}^{{\dot{K}}_{air}}$ for conditions where anode/filter combination of the beam is different than the selection in the software (presented in the legend) are shown as a function of half-value layer of the beam *HVL*_*b*_. The horizontal dashed lines represent ± 5%.

**Fig. 2:**
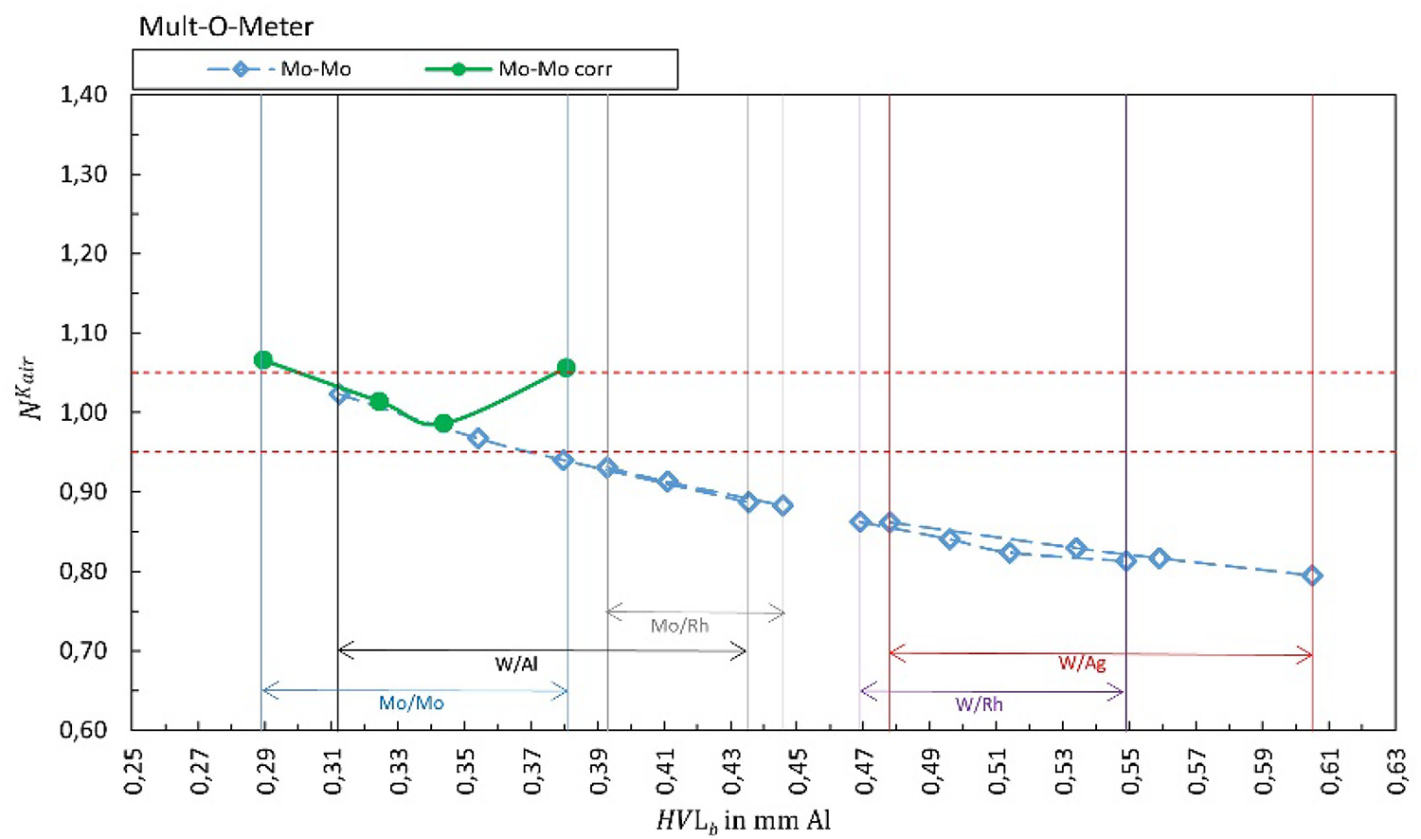
Calibration coefficient for air kerma rate for Mult-O-Meter. The solid green markers represent ${N}_{{X/X}_{b}{=Y/Y}_{s}}^{{\dot{K}}_{air}}$. Open markers represent the calibration coefficients ${N}_{{X/X}_{b}{\ne Y/Y}_{s}}^{{\dot{K}}_{air}}$ for conditions where anode/filter combination of the beam is different than the selection in the software (presented in the legend) are shown as a function of half-value layer of the beam *HVL*_*b*_. The horizontal dashed lines represent ± 5%

**Fig. 3:**
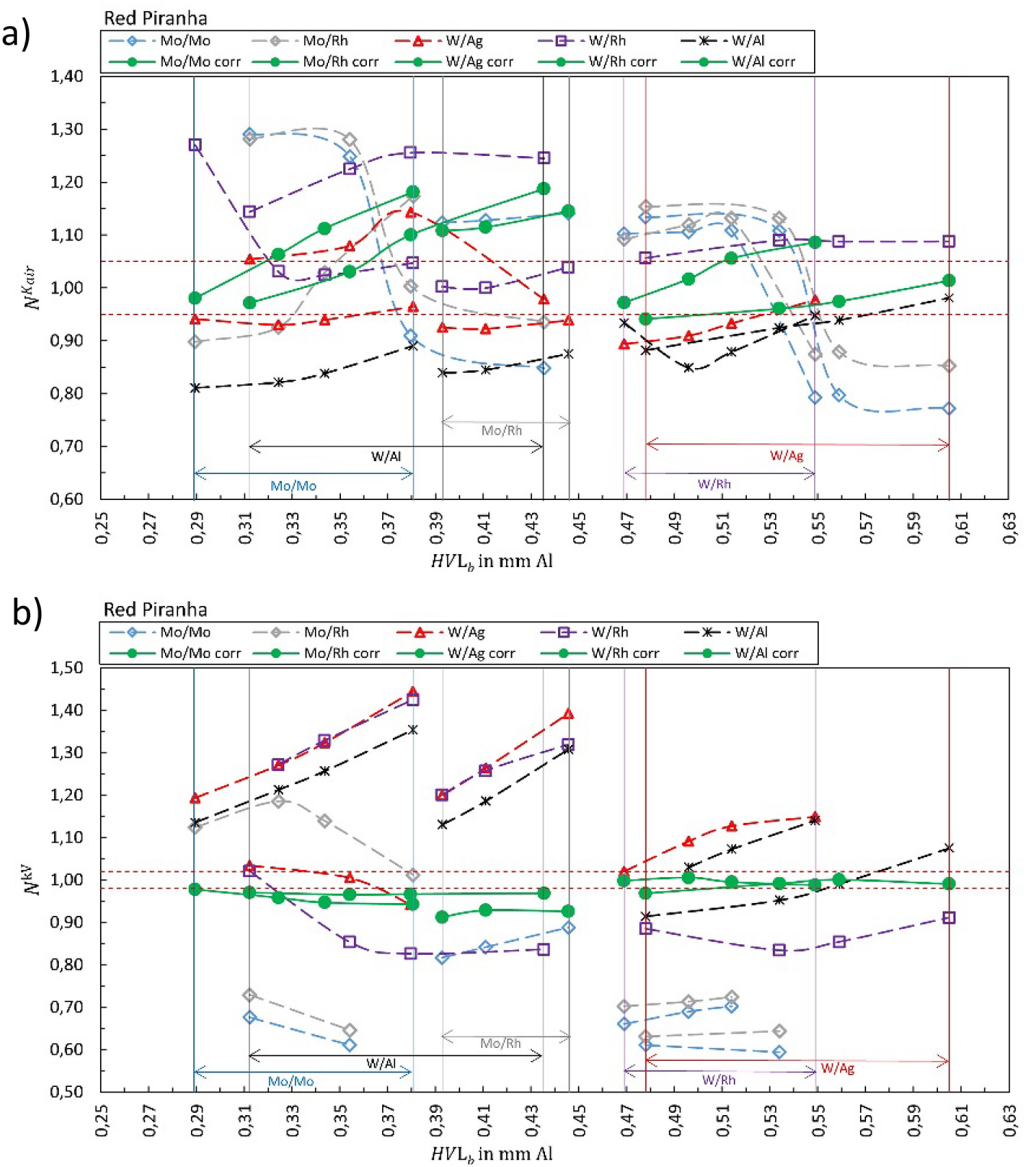
Calibration coefficient ${N}_{{X/X}_{b}{,Y/Y}_{s}}^{{\dot{K}}_{air}kV}$ for (**a**) air kerma rate and (**b**) tube voltage measured by Red Piranha. The solid green markers represent the corresponding correct selection for the anode/filter combination (${X/X}_{b}={Y/Y}_{s}$). Open markers represent the calibration coefficient ${N}_{{X/X}_{b}{,Y/Y}_{s}}$ for conditions where anode/filter combination of the beam is different than the selection in the software (presented in the legend) shown as a function of half-value layer of the beam *HVL*_*b*_. The horizontal dashed lines represent ± 5% and ± 2% for air kerma and kV, respectively.

**Fig. 4:**
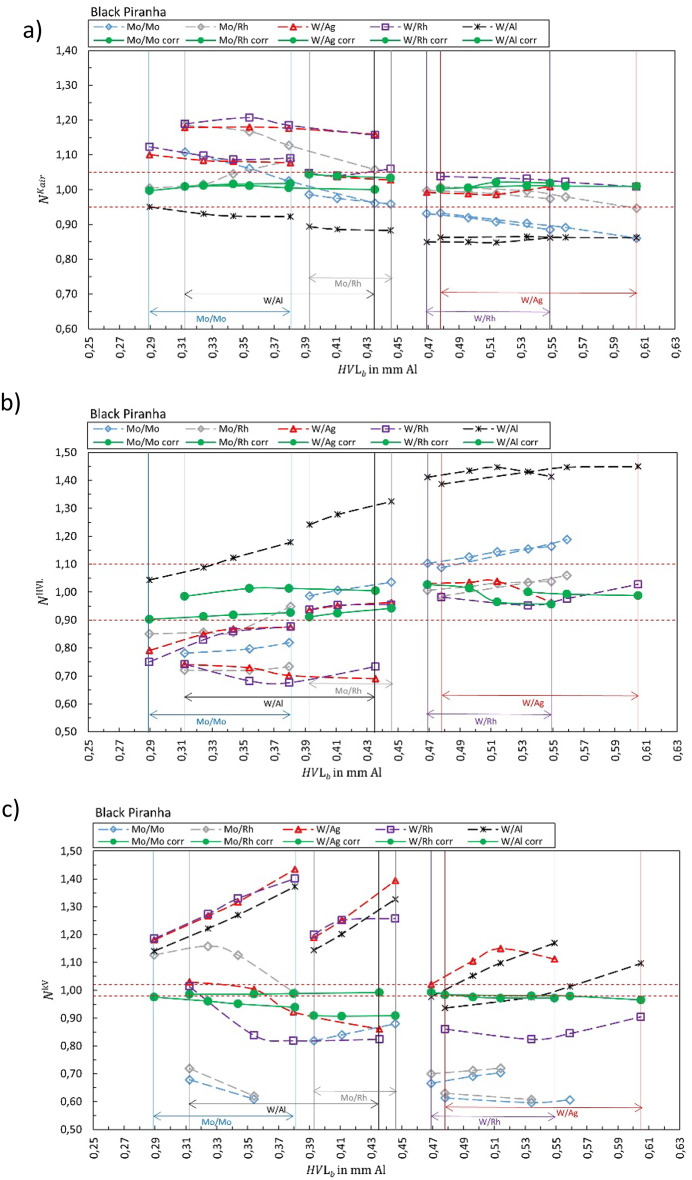
Calibration coefficient ${N}_{{X/X}_{b}{,Y/Y}_{s}}^{{\dot{K}}_{air}kV}$ for (**a**) air kerma rate, (**b**) HVL and (**c**) tube voltage for Black Piranha. The solid green markers represent the corresponding correct selection for the anode/filter combination (${X/X}_{b}={Y/Y}_{s}$). Open markers represent the calibration coefficient ${N}_{{X/X}_{b}{,Y/Y}_{s}}$ for conditions where anode/filter combination of the beam is different than the selection in the software (presented in the legend) shown as a function of half-value layer of the beam *HVL*_*b*_. The horizontal dashed lines represent ± 5%, ± 10%, and ± 2% for air kerma, HVL, and kV, respectively.

**Fig. 5:**
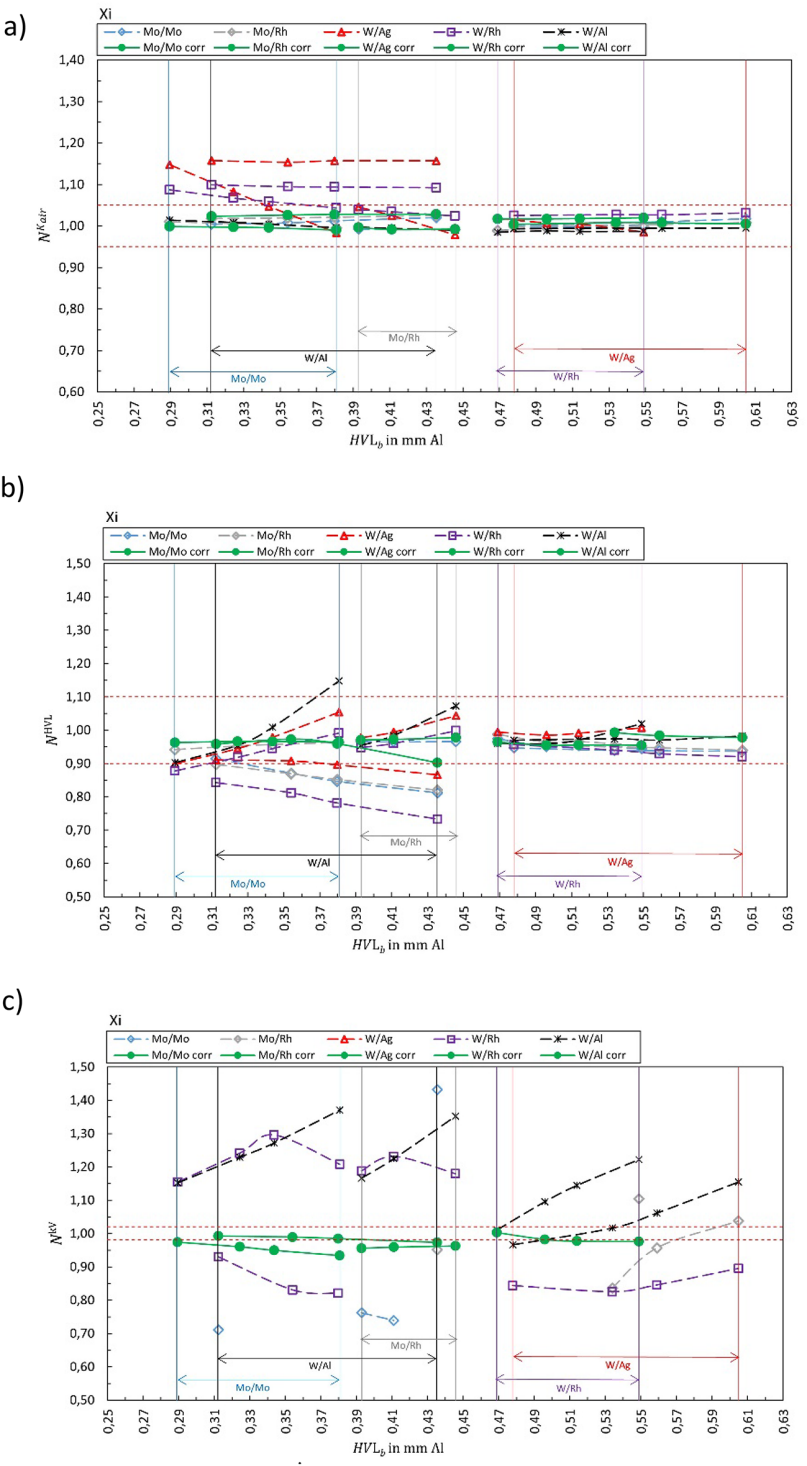
Calibration coefficient ${N}_{{X/X}_{b}{,Y/Y}_{s}}^{{\dot{K}}_{air}kV}$ for (**a**) air kerma rate, (**b**) HVL and (**c**) tube voltage for Xi. The solid green markers represent the corresponding correct selection for the anode/filter combination (${X/X}_{b}={Y/Y}_{s}$). Open markers represent the calibration coefficient ${N}_{{X/X}_{b}{,Y/Y}_{s}}$ for conditions where anode/filter combination of the beam is different than the selection in the software (presented in the legend) shown as a function of half-value layer of the beam *HVL*_*b*_. The horizontal dashed lines represent ± 5%, ± 10%, and ± 2% for air kerma, HVL, and kV, respectively.

**Fig. 6:**
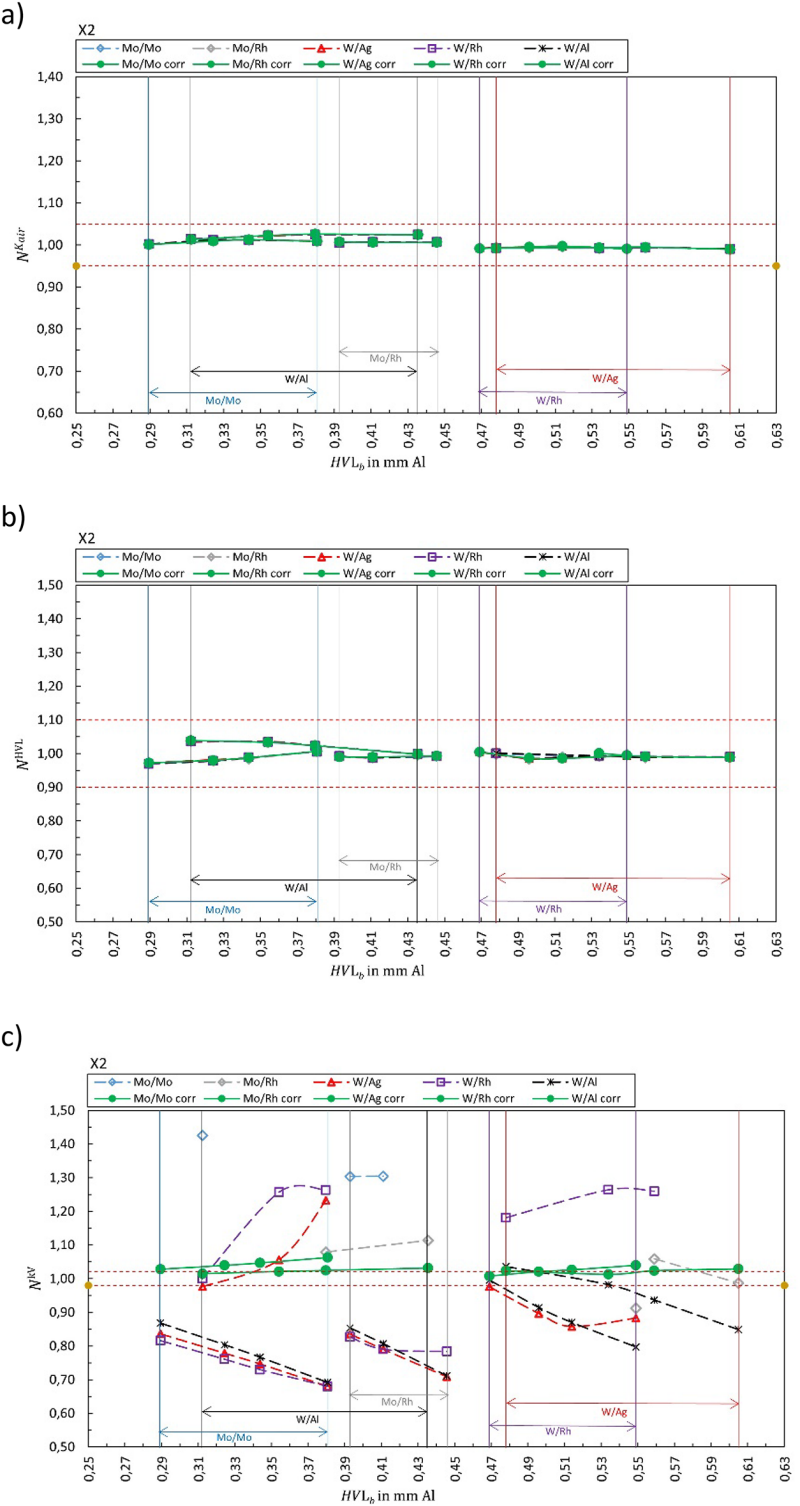
Calibration coefficient ${N}_{{X/X}_{b}{,Y/Y}_{s}}^{{\dot{K}}_{air}kV}$ for (**a**) air kerma rate, (**b**) HVL and (**c**) tube voltage for X2. The solid green markers represent the corresponding correct selection for the anode/filter combination (${X/X}_{b}={Y/Y}_{s}$). Open markers represent the calibration coefficient ${N}_{{X/X}_{b}{,Y/Y}_{s}}$ for conditions where anode/filter combination of the beam is different than the selection in the software (presented in the legend) shown as a function of half-value layer of the beam *HVL*_*b*_. The horizontal dashed lines represent ± 5%, ± 10%, and ± 2% for air kerma, HVL, and kV, respectively.

**Fig. 7:**
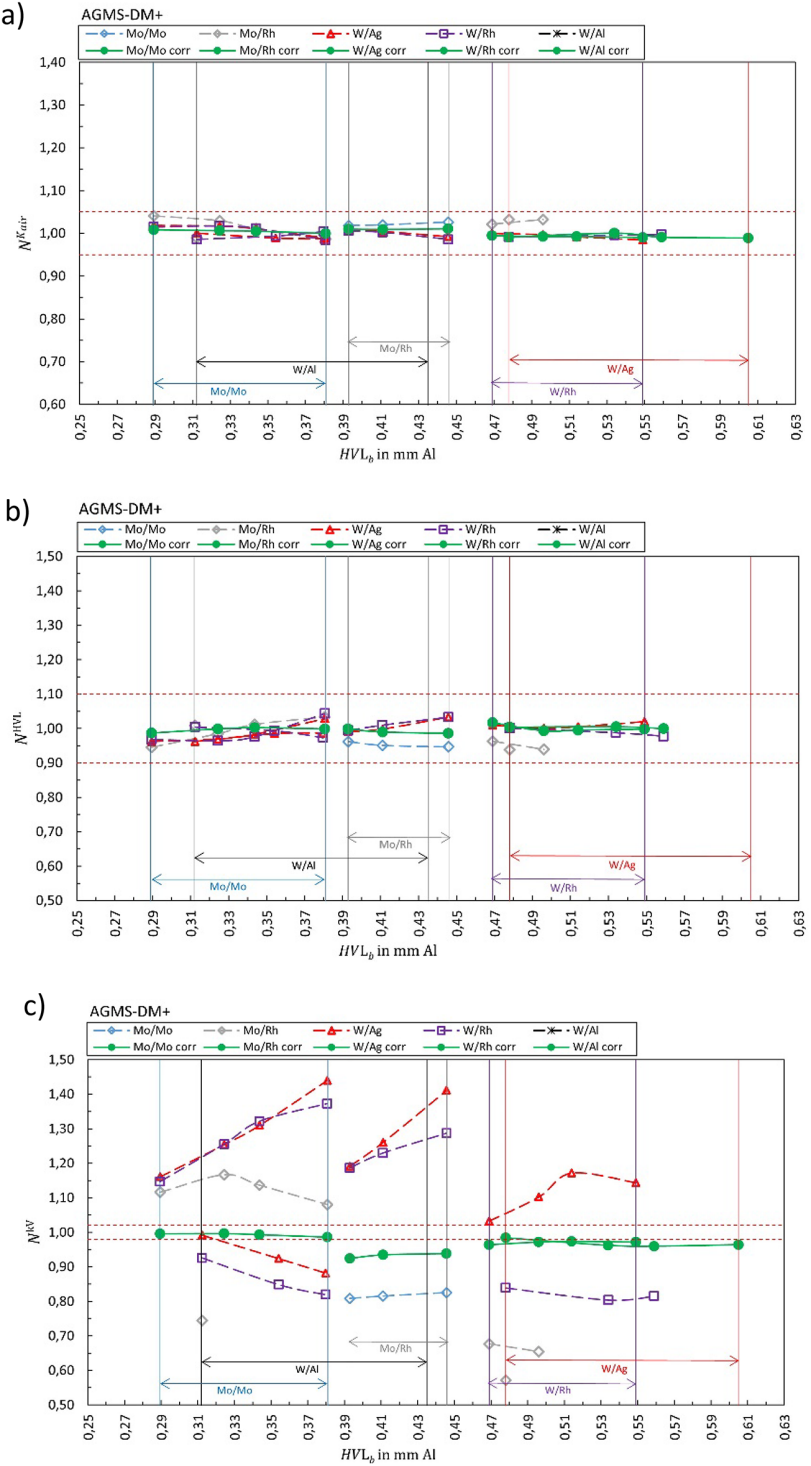
Calibration coefficient ${N}_{{X/X}_{b}{,Y/Y}_{s}}^{{\dot{K}}_{air}kV}$ for (**a**) air kerma rate, (**b**) HVL and (**c**) tube voltage for AGMS-DM+. The solid green markers represent the corresponding correct selection for the anode/filter combination (${X/X}_{b}={Y/Y}_{s}$). Open markers represent the calibration coefficient ${N}_{{X/X}_{b}{,Y/Y}_{s}}$ for conditions where anode/filter combination of the beam is different than the selection in the software (presented in the legend) shown as a function of half-value layer of the beam *HVL*_*b*_. The horizontal dashed lines represent ± 5%, ± 10%, and ± 2% for air kerma, HVL, and kV, respectively.

**Fig. 8:**
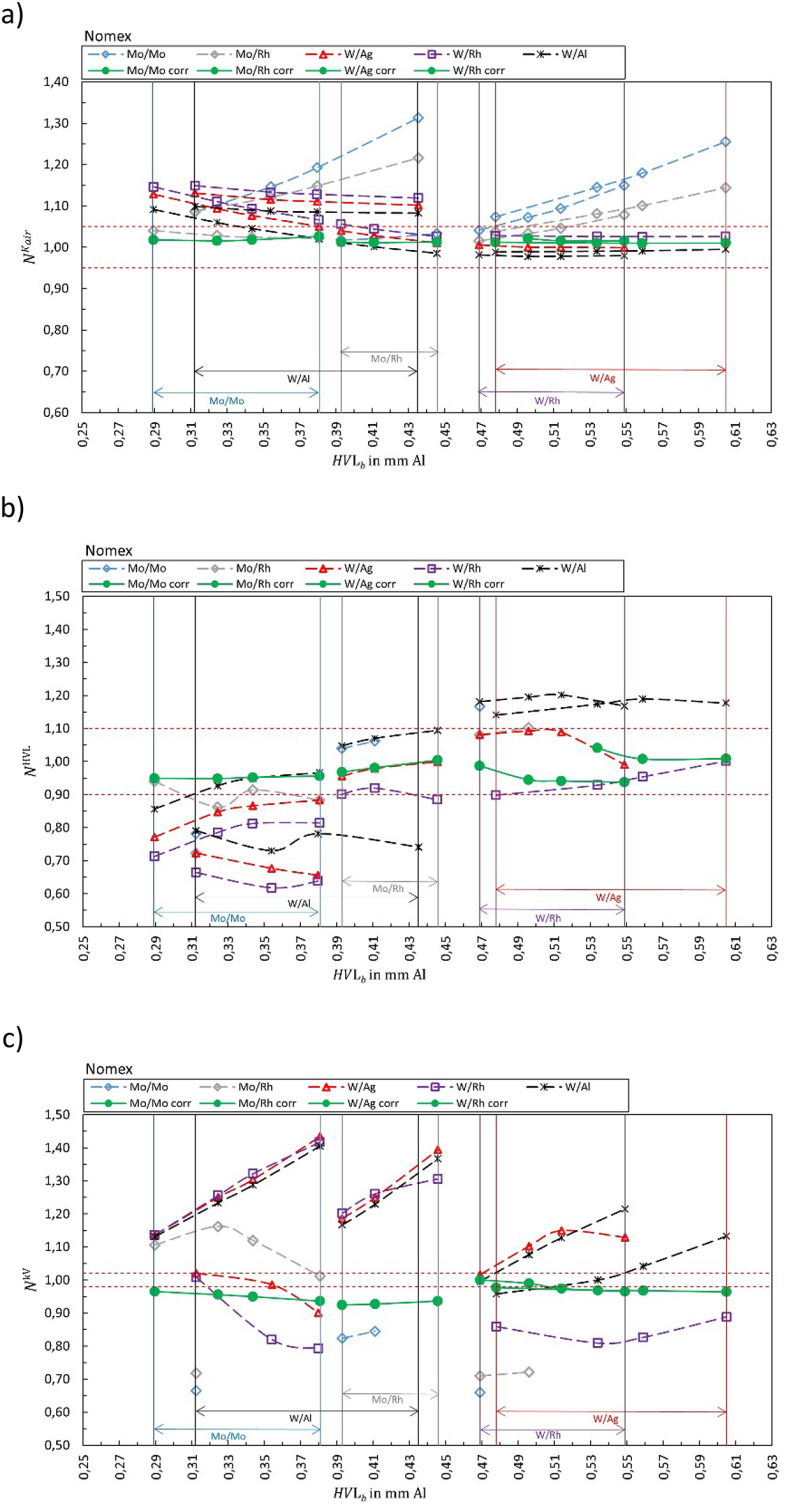
Calibration coefficient ${N}_{{X/X}_{b}{,Y/Y}_{s}}^{{\dot{K}}_{air}kV}$ for (**a**) air kerma rate, (**b**) HVL and (**c**) tube voltage for Nomex. The solid green markers represent the corresponding correct selection for the anode/filter combination (${X/X}_{b}={Y/Y}_{s}$). Open markers represent the calibration coefficient ${N}_{{X/X}_{b}{,Y/Y}_{s}}$ for conditions where anode/filter combination of the beam is different than the selection in the software (presented in the legend) shown as a function of half-value layer of the beam *HVL*_*b*_. The horizontal dashed lines represent ± 5%, ± 10%, and ± 2% for air kerma, HVL, and kV, respectively.

Figures 1, 2, 3, 4, 5, 6, 7, 8 show the calibration coefficient ${N}_{{X/X}_{b}{,Y/Y}_{s}}^{{\dot{K}}_{air},HVL,kV}$ for air kerma (panels a), HVL (panels b), and tube voltage (panels c), respectively, if the quantities are provided by the XMM as a function of HVL of the X-ray beam. The solid green markers represent the corresponding correct selection for the anode/filter combination (${X/X}_{b}={Y/Y}_{s}$). Open markers represent the calibration coefficient for ${X/X}_{b}\ne {Y/Y}_{s}$. The horizontal dashed lines represent ± 5% for air kerma, ± 10% for HVL and ± 2% for tube voltage as the limits stated by most of the manufacturers.

Figure 1 summarizes the results for the R100B, Fig. 2 for the Mult-O-Meter, Fig. 3 for the Red Piranha, Fig. 4 for the Black Piranha, Fig. 5 for the Xi, Fig. 6 for the X2, Fig. 7 for the AGMS-DM+ and Fig. 8 for the Nomex.

The tested XMMs exhibit significant variations in performance and the deviation of the XMMs’ reading to the reference value increases when the wrong anode/filter combination is selected in the software. The R100B and Mult-O-Meter (Figs. 1 and 2) show a distinct energy dependence in their response, whereas most of the other XMMs do not follow a clear pattern. Notably, the X2 and AGMS-DM+ (Figs. 6 and 7) demonstrate a flat energy dependence for both air kerma and HVL measurements. However, for tube voltage measurements, the selection of the anode/filter combination impacts the results.

## Discussion

The results of this study are consistent with those reported by Kojic et al.^21^ who evaluated four XMMs, three of which are also included in the present work, using both a calibration setup and clinical mammography systems. R100B and Xi were tested under ${\text{X}/\text{X}}_{\text{b}}=\text{W}/\text{Al}$ and $\text{Y}/{\text{Y}}_{\text{s}}=\text{Mo}/\text{Mo}$ conditions. The R100B exhibited a pronounced energy dependence, whereas the Xi demonstrated a relatively flat response. Similar, Black Piranha, tested with ${\text{X}/\text{X}}_{\text{b}}=\text{Y}/{\text{Y}}_{\text{s}}=\text{W}/\text{Al}$ also showed a flat response. Costa de Castro et al.^12^ investigated a comparable setup, evaluating Xi, AGMS-DM+, and R100B with ${\text{X}/\text{X}}_{\text{b}}=\text{W}/\text{Al}$ and $\text{Y}/{\text{Y}}_{\text{s}}=\text{Mo}/\text{Mo}$. Their findings indicated errors exceeding 10% when an incorrect anode/filter combination was selected, consistent with the observations in this study. However, their results did not provide such an extensive insight into the expected behavior for a larger range of incorrect selection conditions as was done in the present work.

The two XMMs with a single anode/filter combination available in their software (R100B, Mult-O-Meter) demonstrate a significant energy dependence of their response for air kerma rate. Nonetheless, their response follows a consistent pattern, allowing for interpolation between calibration coefficients with reasonable uncertainty. For XMMs with multiple selections in the software, the correction for air kerma rate is most effective, but there are challenges with tube voltage and HVL measurements.

For the selected anode/filter combination corresponding to the anode/filter combination of the beam, five out of the eight XMMs tested remained within 5% for air kerma rate measurements, while two (R100B, Red Piranha) exceeded 10%. For tube voltage and HVL measurements, all tested XMMs stayed within 10%. In most cases, the calibration coefficient remains reasonably flat or follows a specific trend when the correct anode/filter combination is selected, and the tube voltage varies within the studied 25–35 kV range, in accordance with Salomon et al.^13^. In these cases, interpolating the calibration coefficient for different tube voltages within the stated uncertainty is feasible. However, incorrect selection can introduce significant errors: up to 31% for air kerma, 44% for tube voltage, and 45% for HVL.

In many cases, a user can easily and accidentally make an incorrect selection of the anode/filter combination, and sometimes no correct selection is available. In both scenarios, it is important to understand when and how this impacts the results. Incorrect determination of air kerma and HVL directly affects the calculation of MGD, as the conversion factors are tabulated based on HVL values. This is particularly critical during the commissioning or adjustment of mammography systems, where inaccuracies can lead to incorrect patient dose determination, or improper system calibration, potentially compromising image quality.

To minimize the risk of user error in clinical measurements, it may be beneficial to enable only selection of anode/filter combinations in the software of the XMM that are actually available on the specific mammography manufacturer and/or system, rather than solely providing a list of anode/filter combinations. Additionally, a significant improvement would be the integration of a warning system in the XMMs, alerting users when the measured X-ray spectrum deviates substantially from the expected spectrum for the selected settings.

Calibration with only one anode/filter combination is exclusively reliable for XMMs when air kerma/air kerma rate and HVL readings are independent of software selection. Although only X2 states that air kerma and HVL measurements are independent of software selection, measurements indicate that this is also largely correct for the AGMS-DM+. For X2, the deviation is a maximum of 0.3% for air kerma and 0.5% for HVL, for AGMS-DM+, the deviation is a maximum of 4% for air kerma and 7% for HVL when comparing incorrect and correct selections. Thus, calibration with incorrect selections has limited impact on the resulting calibration coefficient. It should be emphasized that these are special cases. In general, for XMMs with multiple software selections, a fixed selection of radiation quality for different beams does not show a clear trend of response, making interpolation for other anode/filter combinations impossible. It could be beneficial to consider a test for the effect of selection as part of calibration tests, in addition to using the correct selection.

The anode/filter combinations for calibrating XMMs for mammography could be updated to better reflect the wide range of clinical combinations. However, most calibration laboratories cannot establish a broad range of clinically representative radiation qualities. Therefore, the metrology community should devise a method to ensure these dosimeters provide reliable results under all conditions. One option is to ensure type testing covers the clinical range, with a software selection available for calibration conditions to verify that the XMMs operate within an acceptable range. Based on the findings, it is not recommended to interpolate between calibration coefficients for different anode/filter combinations. However, small interpolation for tube voltage within the calibration range could be considered acceptable.

It is important to note that calibration is typically performed without the additional filtration introduced by a compression plate, whereas clinical measurements are usually conducted with a compression plate in place. This additional filtration alters the X-ray spectrum and can impact measurement accuracy. While uncertainties associated with minor variations in radiation quality, such as those caused by the compression plate may be acceptable, their contribution to the overall uncertainty should be carefully considered^12^.

Further research is recommended to develop standardized calibration protocols that cover the full spectrum of clinically relevant anode/filter combinations utilized in mammography. This research could include an extensive evaluation of the performance XMMs on mammography systems, with the aim of correlating their behavior under calibration conditions to their performance under clinical conditions for which they are optimized. This would also provide relevant data on the impact of changes in measurement conditions, which is needed for uncertainty analysis. Based on these findings, calibration protocols can be refined and adapted to enhance their accuracy and clinical applicability.

## Conclusions

Modern dosimetry devices with a range of radiation quality selection and software-based corrections provide improved accuracy for measurement of air kerma, tube voltage and HVL with different radiation qualities. However, an appropriate selection of the anode/filter combination in the XMM software is crucial for obtaining reliable measurement results. Interpolation of calibration coefficients between different radiation qualities and selections is not recommended. Therefore, it is practically impossible to have a comprehensive coverage of traceability for all selections and measurements conditions used in clinical practice. This emphasizes the role of type testing and the need to define such a calibration procedure which can be established and routinely used in national calibration laboratories to ensure proper functioning of XMMs in all clinical conditions.

## Data Availability

The datasets generated and analysed during the current study are available in the Zenodo repository, [10.5281/zenodo.13304517]. Alternatively, the dataset can also be requested from the corresponding author via this e-mail: elisabeth.salomon@meduniwien.ac.at.
